# Facile Synthesis and Characterizations of Mixed Metal Oxide Nanoparticles for the Efficient Photocatalytic Degradation of Rhodamine B and Congo Red Dyes

**DOI:** 10.3390/nano12223992

**Published:** 2022-11-12

**Authors:** Ehab A. Abdelrahman, Eida S. Al-Farraj

**Affiliations:** 1 Department of Chemistry, College of Science, Imam Mohammad Ibn Saud Islamic University (IMSIU), Riyadh 11623, Saudi Arabia; 2 Chemistry Department, Faculty of Science, Benha University, Benha 13518, Egypt

**Keywords:** Fe_0.5_Mn_0.5_Co_2_O_4_/Fe_2_O_3_, Mn_0.5_Zn_0.5_Fe_2_O_4_/Fe_2_O_3_, Pechini sol–gel method, Rhodamine B dye, Congo Red, photocatalytic degradation

## Abstract

Photocatalytic degradation has been suggested to be a cheap and efficient way to dispose of organic pollutants, such as dyes. Therefore, our research team strives to produce nanophotocatalysts in a simple and inexpensive way. In this work, the Pechini sol–gel technique was employed for the facile synthesis of Mn_0.5_Zn_0.5_Fe_2_O_4_/Fe_2_O_3_ and Fe_0.5_Mn_0.5_Co_2_O_4_/Fe_2_O_3_ as mixed metal oxide nanoparticles for the efficient photocatalytic degradation of Rhodamine B and Congo Red dyes. XRD, FT-IR, a N_2_ adsorption/desorption analyzer, EDS, FE-SEM, and an UV–Vis diffuse reflectance spectrophotometer were used to characterize the produced samples. The XRD patterns revealed that the average crystallite size of the Fe_0.5_Mn_0.5_Co_2_O_4_/Fe_2_O_3_ and Mn_0.5_Zn_0.5_Fe_2_O_4_/Fe_2_O_3_ samples is 90.25 and 80.62 nm, respectively. The FE-SEM images revealed that the Fe_0.5_Mn_0.5_Co_2_O_4_/Fe_2_O_3_ sample consists of cubic and irregular shapes with an average diameter of 1.71 µm. Additionally, the Mn_0.5_Zn_0.5_Fe_2_O_4_/Fe_2_O_3_ sample consists of spherical shapes with an average diameter of 0.26 µm. The energy gaps of the Fe_0.5_Mn_0.5_Co_2_O_4_/Fe_2_O_3_ and Mn_0.5_Zn_0.5_Fe_2_O_4_/Fe_2_O_3_ samples are 3.50 and 4.3 eV and 3.52 and 4.20 eV, respectively. In the presence of hydrogen peroxide, the complete degradation of 100 mL of 20 mg/L of Rhodamine B and Congo Red dyes occurred at pH = 8 and 3, respectively, within 50 min, using 0.1 g of the synthesized samples.

## 1. Introduction

Organic dyes are present in water sources due to their many industrial uses, such as in paper, textiles, food, cosmetics, and plastics. Because they can cause cancer and make cells change, these dye molecules are harmful to living things. Throwing liquid waste containing dyes into water leads to severe health risks for humans [1,2,3,4,5]. Therefore, effective strategies must be found to dispose of these pollutants.

Many methods are used to remove these pollutants, such as microfiltration, precipitation, reverse osmosis, chemical coagulation, photocatalytic degradation, and adsorption [6,7,8,9,10,11,12,13,14,15]. The recently arising solar-driven interfacial evaporation and photocatalysis method is a new strategy to remove organic pollutants from water with catalysts at the air–water interface [16,17,18].

Photocatalytic degradation has been suggested to be a cheap and efficient way to dispose of dye molecules. The absorption of photons by a photocatalyst results in the transmission of some electrons from the valence band to the conduction band. Hence, this simultaneously generates electrons and holes in the conduction and valence bands, respectively. Electrons and holes can produce hydroxyl free radicals when reacting with water. Organic dye pollutants can be quickly degraded by hydroxyl free radicals and converted into volatile gases, such as CO_2_ and H_2_O [19,20].

Congo Red and Rhodamine B dyes are utilized in numerous industries, such as the textile, chemical, pharmaceutical, paper, and cosmetic sectors. Consequently, a substantial quantity of these compounds contaminate water and enter normal water foundations. Due to the presence of aromatic amines in the composition of these compounds, ingestion of these dyes can cause cancer. This is why environmental rules require enterprises to remove these harmful compounds from polluted industrial fluids before releasing them into the environment [21,22,23].

There are many nanomaterials that are used as catalysts for the degradation of many organic dyes, such as CuO/TiO_2_/VO_2_, MgAlTi, ZnAlTi, perovskites, graphene oxide/silver nanocomposites, ZnO/montmorillonite nanocomposites, TiO_2_-impregnated activated carbon, titanium oxo ethoxo clusters, nitrogen-doped TiO_2_ nanotubes, and TiO_2_/Fe_2_O_3_ nanocomposites [24,25,26,27,28,29,30,31,32]. Nanomaterials are characterized by a large surface area, high stability, and high efficiency in producing free radicals.

Several methods have been used to synthesize nanomaterials with a wide range of morphologies and sizes. The Pechini sol–gel method is widely used in the preparation of many nanomaterials, such as CdTiO_3_, Y_2_O_3_, SrTiO_3_, and BaTiO_3_ [33,34,35,36]. However, most of the chemicals used to prepare these materials are very expensive. Therefore, our research team strives to produce nanophotocatalysts in a simple and inexpensive way.

Hence, in this work, the Pechini sol–gel technique was utilized for the facile synthesis of Mn_0.5_Zn_0.5_Fe_2_O_4_/Fe_2_O_3_ and Fe_0.5_Mn_0.5_Co_2_O_4_/Fe_2_O_3_ as mixed metal oxide nanoparticles for the efficient photocatalytic degradation of Rhodamine B and Congo Red dyes. The first innovative aspect of our research comes from the use of cheap salts, such as cobalt(II) chloride hexahydrate, iron(III) chloride hexahydrate, and manganese(II) acetate tetrahydrate to obtain these mixed nano-oxides for the first time. The other innovative aspect of our research comes from the ability of these nanomaterials to degrade a large concentration and a large volume of the Congo Red and Rhodamine B dyes in a short time. XRD, FT-IR, a N_2_ adsorption/desorption analyzer, EDS, field-emission scanning electron microscopy, transmission electron microscopy, and an UV–Vis diffuse reflectance spectrophotometer were used to characterize the produced samples. In addition, the factors impacting the degradation of Rhodamine B and Congo Red dyes, such as pH, time, concentration, and catalyst quantity, were investigated.

## 2. Experimental Section

### 2.1. Chemicals

Ethylene glycol (C_2_H_6_O_2_), cobalt(II) chloride hexahydrate (CoCl_2_.6H_2_O), hydrogen peroxide (H_2_O_2_), iron(III) chloride hexahydrate (FeCl_3_.6H_2_O), hydrochloric acid (HCl), manganese(II) acetate tetrahydrate (Mn(CH_3_COO)_2_.4H_2_O), zinc(II) sulfate heptahydrate (ZnSO_4_.7H_2_O), sodium hydroxide (NaOH), Rhodamine B dye (C_28_H_31_ClN_2_O_3_), Congo Red dye (C_32_H_22_N_6_Na_2_O_6_S_2_), and tartaric acid (C_4_H_6_O_6_) were purchased from Sigma Aldrich Company and used as received without further purification.

### 2.2. Synthesis of Mn_0.5_Zn_0.5_Fe_2_O_4_/Fe_2_O_3_ and Fe_0.5_Mn_0.5_Co_2_O_4_/Fe_2_O_3_ Samples

**Scheme 1:**
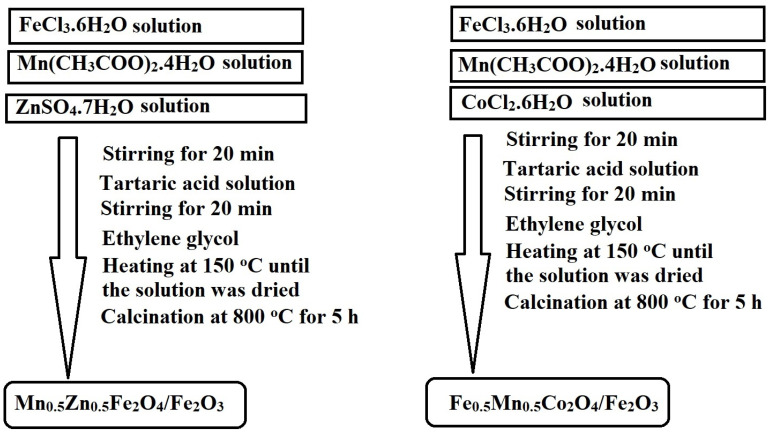
The synthesizing steps of the nanomaterials.

In 60 mL of distilled water, 8.33 g of FeCl_3_.6H_2_O, 4.43 g of ZnSO_4_.7H_2_O, 3.79 g of Mn(CH_3_COO)_2_.4H_2_O, and 1.47 g of CoCl_2_.6H_2_O were dissolved separately. Additionally, 13.25 g of tartaric acid was dissolved in 60 mL of distilled water. Fe(III), Zn(II), and Mn(II) solutions were mixed and stirred for 20 min to create the Mn_0.5_Zn_0.5_Fe_2_O_4_/Fe_2_O_3_ product. Moreover, Fe(III), Co(II), and Mn(II) solutions were mixed and stirred for 20 min to create the Fe_0.5_Mn_0.5_Co_2_O_4_/Fe_2_O_3_ product. Afterward, a tartaric acid solution was added to each system with continuous stirring for 20 min. Additionally, 3.33 mL of ethylene glycol was added to each system with continuous stirring. Furthermore, each system was heated at 150 °C until the solution was dried. Finally, the formed powder was ignited at 800 °C for 5 h. Scheme 1 summarizes the synthesizing steps of the nanomaterials.

### 2.3. Instrumentation

An X-ray diffraction (XRD) instrument was used to study the crystalline structure of the synthesized nanomaterials. The diffractograms were collected using a D8 Advance X-ray diffractometer equipped with K_α_Cu radiation (λ = 0.15 nm). A Thermo Scientific Nicolet iS50 Fourier-transform infrared spectrometer (FT-IR) was used to study the functional groups of the synthesized nanomaterials. A Jasco V-750 UV–Vis diffuse reflectance spectrophotometer (DRS) and an integrating sphere, calibrated with barium sulfate, were used to determine the band gap of the synthesized nanomaterials. A Quantachrome NOVA Touch LX2 nitrogen-gas-sorption analyzer was used to study the surface textures (BET surface area, total pore volume, and average pore radius) of the synthesized nanomaterials. The synthesized nanomaterials were degassed at 110 °C for 24 h before analyses. A Quanta 250 FEG scanning electron microscope (SEM) attached with an energy dispersive X-ray unit was used to study the surface morphology and elemental analysis of the synthesized nanomaterials. The morphologies of the nanomaterials were obtained using a Talos F200iS transmission electron microscope (TEM). The concentration of Rhodamine B and Congo Red dyes was determined using a Jasco V-750 UV–Vis spectrophotometer. The maximum wavelengths of the Rhodamine B and Congo Red dyes were 554 and 497 nm, respectively.

### 2.4. Photocatalytic Degradation of Rhodamine B and Congo Red Dyes

For every experiment, a specified amount of the Mn_0.5_Zn_0.5_Fe_2_O_4_/Fe_2_O_3_ or Fe_0.5_Mn_0.5_Co_2_O_4_/Fe_2_O_3_ samples was dispersed in a 100 mL aqueous solution of Rhodamine B or Congo Red dyes. The suspension was then agitated magnetically in the dark for 60 min. The solution was then irradiated with three UV lamps (30 cm, 8 watt, and 225 nm) located 8 cm away from the dye solution. In addition, the nanomaterials were separated by centrifugation, and the remaining concentration of the Rhodamine B or Congo Red dyes in the filtrate was measured using a Jasco V-750 UV–Vis spectrophotometer. The same tests were conducted again, but this time 2 mL of 2 M hydrogen peroxide was added. The photodegradation efficiency (% D) of the nanomaterials against Rhodamine B or Congo Red dyes was determined using Equation (1).

(1) $$\%\;D=\frac{X_{d}-X_{e}}{X_{d}}\times 100$$

*X_d_* (mg/L) is the remaining concentration of the Rhodamine B or Congo Red dyes after the process of stirring in the dark. *X_e_* (mg/L) is the remaining concentration of the Rhodamine B or Congo Red dyes after exposure to ultraviolet rays.

## 3. Results and Discussion

### 3.1. Characterization of the Synthesized Nanocomposites

#### 3.1.1. X-ray Diffraction

**Figure 1:**
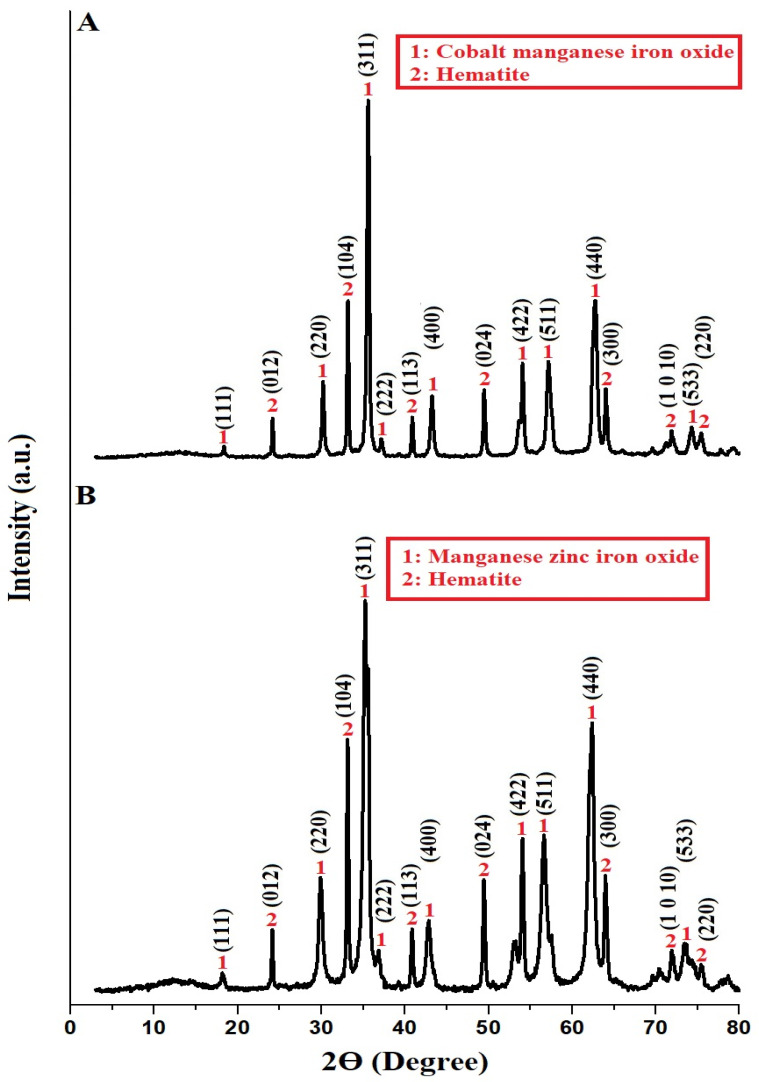
The X-ray diffraction patterns of the Fe_0.5_Mn_0.5_Co_2_O_4_/Fe_2_O_3_ (**A**) and Mn_0.5_Zn_0.5_Fe_2_O_4_/Fe_2_O_3_ (**B**) samples.

X-ray powder diffraction (XRD) is a rapid analytical technique primarily used for the phase identification of a crystalline material, and it can provide information on the average crystallite size. Figure 1A,B displays the X-ray diffraction patterns of the Fe_0.5_Mn_0.5_Co_2_O_4_/Fe_2_O_3_ and Mn_0.5_Zn_0.5_Fe_2_O_4_/Fe_2_O_3_ samples, respectively. The results reveal that the Fe_0.5_Mn_0.5_Co_2_O_4_/Fe_2_O_3_ sample consisted of hematite (Fe_2_O_3_) and cobalt manganese iron oxide (Fe_0.5_Mn_0.5_Co_2_O_4_), as indicated by JCPDS Nos. 00-024-0072 and 01-086-8898, respectively. Additionally, the Mn_0.5_Zn_0.5_Fe_2_O_4_/Fe_2_O_3_ sample consisted of hematite (Fe_2_O_3_) and manganese zinc iron oxide (Mn_0.5_Zn_0.5_Fe_2_O_4_), as indicated by JCPDS Nos. 00-024-0072 and 01-086-8880, respectively. The peaks of cobalt manganese iron oxide or manganese zinc iron oxide at 2Ɵ = 74.17°, 62.66°, 57.16°, 53.85°, 43.38°, 37.06°, 35.45°, 30.10°, and 18.38° corresponded to lattice plans (533), (440), (511), (422), (400), (222), (311), (220), and (111), respectively, as obtained from JCPDS Nos. 01-086-8898 and 01-086-8880. The peaks of hematite at 2Ɵ = 75.40°, 71.83°, 64.01°, 49.54°, 40.84°, 33.14°, and 24.07° corresponded to lattice plans (220), (1 0 10), (300), (024), (113), (104), and (012), respectively, as obtained from JCPDS No. 00-024-0072. The average crystallite size of the Fe_0.5_Mn_0.5_Co_2_O_4_/Fe_2_O_3_ and Mn_0.5_Zn_0.5_Fe_2_O_4_/Fe_2_O_3_ samples was 90.25 and 80.62 nm, respectively. This confirms the success of the Pechini sol–gel method in synthesizing new mixed nano-oxides. In this method, an aqueous solution of metal salts is mixed with tartaric acid. Chelation, or the formation of complex ring-shaped compounds around the metal cations, takes place in the solution. Ethylene glycol is then added, and the liquid is heated to 150 °C to allow the chelates to polymerize, or form large, cross-linked networks. As excess water is removed by heating, a solid polymeric resin is achieved. Eventually, at a higher temperature of 800 °C for 5 h, the resin is decomposed, and ultimately, mixed metal oxides are obtained. Hence, this explains the proportions of the elements in Table 1 [33].

#### 3.1.2. Energy Dispersive X-ray Spectroscopy

**Figure 2:**
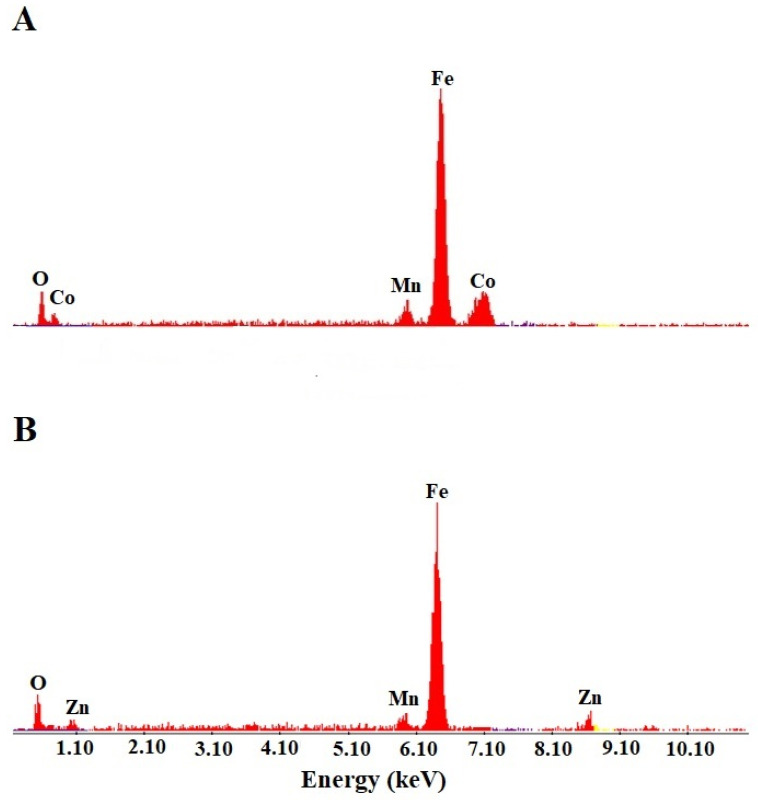
The energy dispersive X-ray patterns of the Fe_0.5_Mn_0.5_Co_2_O_4_/Fe_2_O_3_ (**A**) and Mn_0.5_Zn_0.5_Fe_2_O_4_/Fe_2_O_3_ (**B**) samples.

Energy dispersive X-ray spectroscopy (EDX) is an analytical method which yields a spectrum that displays the peaks correlated to the elemental composition of the investigated sample. Figure 2A,B displays the energy dispersive X-ray patterns of the Fe_0.5_Mn_0.5_Co_2_O_4_/Fe_2_O_3_ and Mn_0.5_Zn_0.5_Fe_2_O_4_/Fe_2_O_3_ samples, respectively. The results reveal that the Fe_0.5_Mn_0.5_Co_2_O_4_/Fe_2_O_3_ sample consisted of manganese, oxygen, cobalt, and iron as displayed in Table 1. Additionally, the Mn_0.5_Zn_0.5_Fe_2_O_4_/Fe_2_O_3_ sample consisted of manganese, oxygen, zinc, and iron as displayed in Table 1. Hence, the absence of other elements confirms the success of the method in obtaining pure nano-oxides. The high percentage of iron in the Fe_0.5_Mn_0.5_Co_2_O_4_/Fe_2_O_3_ sample confirms the high percentage of Fe_2_O_3_ compared to Fe_0.5_Mn_0.5_Co_2_O_4_.

#### 3.1.3. N_2_ Adsorption/Desorption Analyzer

**Figure 3:**
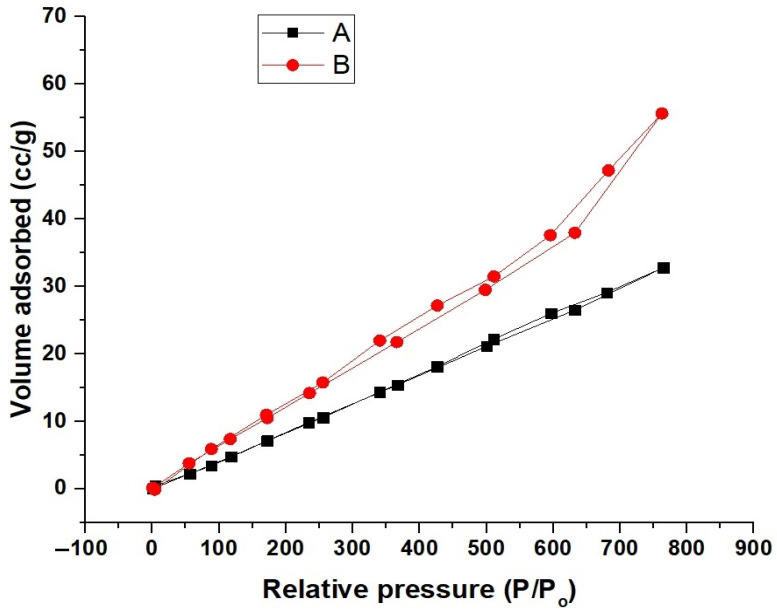
The plot of the volume adsorbed against the relative pressure for the Fe_0.5_Mn_0.5_Co_2_O_4_/Fe_2_O_3_ (A) and Mn_0.5_Zn_0.5_Fe_2_O_4_/Fe_2_O_3_ (B) samples.

Figure 3A,B displays the plot of the volume adsorbed against the relative pressure of the Fe_0.5_Mn_0.5_Co_2_O_4_/Fe_2_O_3_ and Mn_0.5_Zn_0.5_Fe_2_O_4_/Fe_2_O_3_ samples, respectively. The results reveal that all curves belong to the IV types [37,38,39]. In addition, Table 2 displays the surface parameters, such as the BET surface area, total pore volume, and average pore size, of the produced samples. Moreover, the BET surface area of the Mn_0.5_Zn_0.5_Fe_2_O_4_/Fe_2_O_3_ sample is greater than that of the Fe_0.5_Mn_0.5_Co_2_O_4_/Fe_2_O_3_ sample. Hence, it was expected that the Mn_0.5_Zn_0.5_Fe_2_O_4_/Fe_2_O_3_ sample would outperform the Fe_0.5_Mn_0.5_Co_2_O_4_/Fe_2_O_3_ sample in the photocatalytic degradation efficiency of the dyes under study.

#### 3.1.4. Field-Emission Scanning Electron Microscopy and Transmission Electron Microscopy

**Figure 4:**
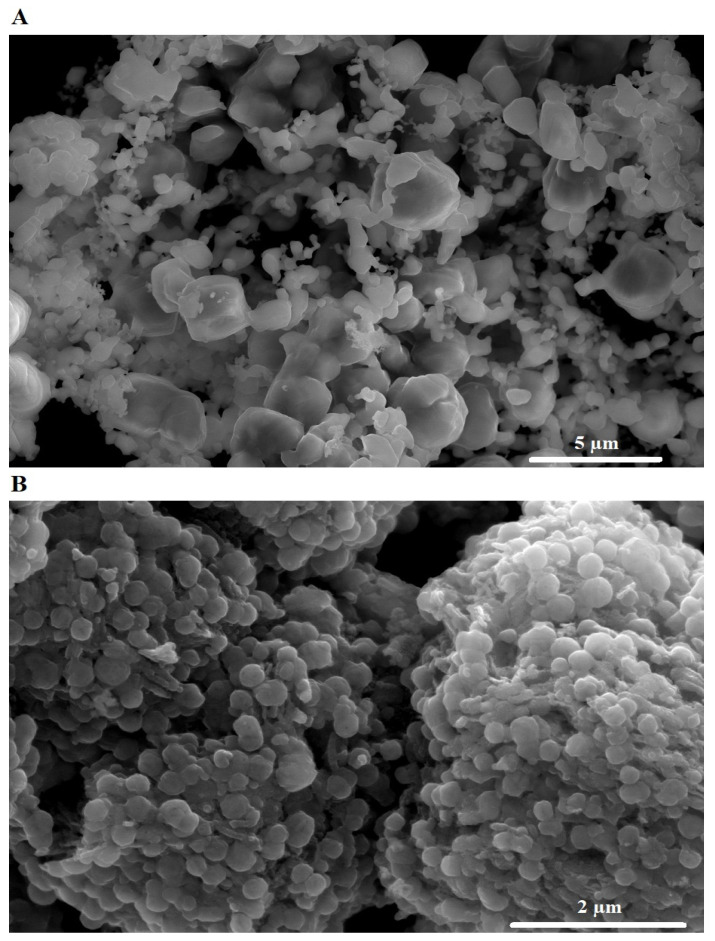
The scanning electron microscopy images of the Fe_0.5_Mn_0.5_Co_2_O_4_/Fe_2_O_3_ (**A**) and Mn_0.5_Zn_0.5_Fe_2_O_4_/Fe_2_O_3_ (**B**) samples.

Figure 4A,B displays the scanning electron microscopy images of the Fe_0.5_Mn_0.5_Co_2_O_4_/Fe_2_O_3_ and Mn_0.5_Zn_0.5_Fe_2_O_4_/Fe_2_O_3_ samples, respectively. The results reveal that the surface of the Fe_0.5_Mn_0.5_Co_2_O_4_/Fe_2_O_3_ sample consisted of cubic and irregular shapes with an average grain size of 1.71 µm. Additionally, the surface of the Mn_0.5_Zn_0.5_Fe_2_O_4_/Fe_2_O_3_ sample consists of spherical shapes with an average grain size of 0.26 µm. XRD can determine the crystallite size, but SEM can determine the grain size, and the surface particles might consist of many aggregates of crystallites, which should be bigger than the crystallite size obtained from XRD.

**Figure 5:**
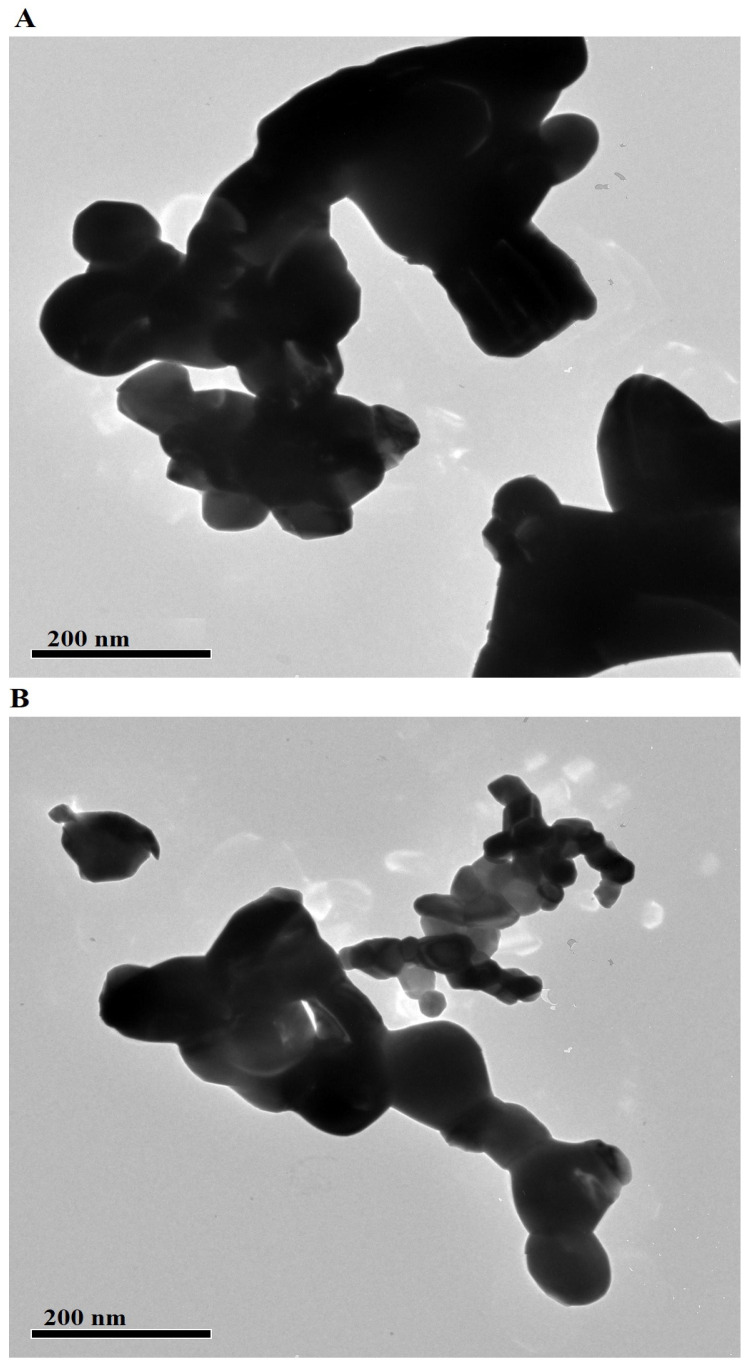
The transmission electron microscopy images of the Fe_0.5_Mn_0.5_Co_2_O_4_/Fe_2_O_3_ (**A**) and Mn_0.5_Zn_0.5_Fe_2_O_4_/Fe_2_O_3_ (**B**) samples.

Figure 5A,B displays the transmission electron microscopy images of the Fe_0.5_Mn_0.5_Co_2_O_4_/Fe_2_O_3_ and Mn_0.5_Zn_0.5_Fe_2_O_4_/Fe_2_O_3_ samples, respectively. The results reveal that the Fe_0.5_Mn_0.5_Co_2_O_4_/Fe_2_O_3_ and Mn_0.5_Zn_0.5_Fe_2_O_4_/Fe_2_O_3_ samples consisted of cubic and irregular shapes with an average diameter of 100.27 and 84.29 nm, respectively. The average particle size determined from the TEM images is slightly larger than that estimated from the XRD technique as a result of the presence of the agglomeration.

#### 3.1.5. FT-IR

**Figure 6:**
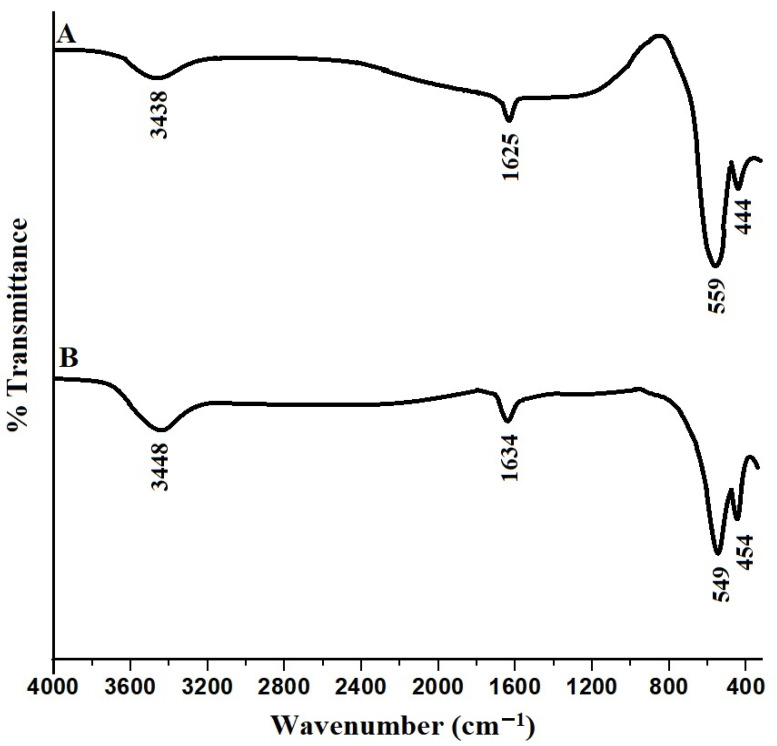
The FT-IR spectra of the Fe_0.5_Mn_0.5_Co_2_O_4_/Fe_2_O_3_ (**A**) and Mn_0.5_Zn_0.5_Fe_2_O_4_/Fe_2_O_3_ (**B**) samples.

Figure 6A,B displays the FT-IR spectra of the Fe_0.5_Mn_0.5_Co_2_O_4_/Fe_2_O_3_ and Mn_0.5_Zn_0.5_Fe_2_O_4_/Fe_2_O_3_ samples, respectively. The bands at 559 and 549 cm^−1^ in the Fe_0.5_Mn_0.5_Co_2_O_4_/Fe_2_O_3_ and Mn_0.5_Zn_0.5_Fe_2_O_4_/Fe_2_O_3_ samples were attributed to the bending vibrational modes of Fe-O, Mn-O, Co-O, and Zn-O in the Fe_0.5_Mn_0.5_Co_2_O_4_ and Mn_0.5_Zn_0.5_Fe_2_O_4_/Fe_2_O_3_ samples, respectively. The bands at 444 and 454 cm^−1^ in the Fe_0.5_Mn_0.5_Co_2_O_4_/Fe_2_O_3_ and Mn_0.5_Zn_0.5_Fe_2_O_4_/Fe_2_O_3_ samples were attributed to the bending vibrations of the Fe–O bond in the Fe_2_O_3_, respectively. The bands at 1625 and 1634 cm^−1^ in the Fe_0.5_Mn_0.5_Co_2_O_4_/Fe_2_O_3_ and Mn_0.5_Zn_0.5_Fe_2_O_4_/Fe_2_O_3_ samples, respectively, were attributed to the bending vibrations of the adsorbed water. The bands at 3433 and 3448 cm^−1^ in the Fe_0.5_Mn_0.5_Co_2_O_4_/Fe_2_O_3_ and Mn_0.5_Zn_0.5_Fe_2_O_4_/Fe_2_O_3_ nanocomposites, respectively, were attributed to the stretching vibrations of the adsorbed water [40].

#### 3.1.6. Energy Gap

The energy gap (E_g_) was determined using the diffuse reflectance spectra of the Fe_0.5_Mn_0.5_Co_2_O_4_/Fe_2_O_3_ and Mn_0.5_Zn_0.5_Fe_2_O_4_/Fe_2_O_3_ samples and Equation (2) [19].

(F(R)hυ)^Z^ = K (hυ − E_g_) (2)

**Figure 7:**
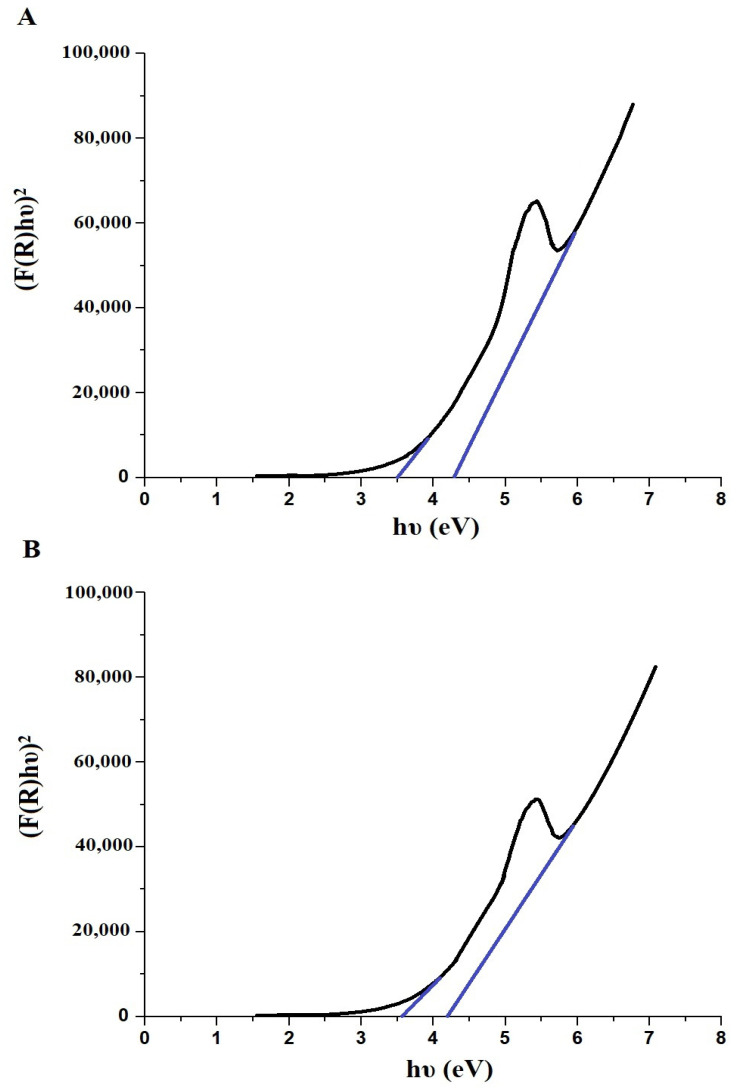
The plot of (F(R)hυ)^2^ against hυ for the Fe_0.5_Mn_0.5_Co_2_O_4_/Fe_2_O_3_ (**A**) and Mn_0.5_Zn_0.5_Fe_2_O_4_/Fe_2_O_3_ (**B**) samples.

F(R) is a constant, while K is the Kubelka–Munk function. Z is an integer based on the transition type. Z = 2 for the direct transitions that are permitted, while Z = 1/2 for the indirect transitions that are permitted. Figure 7A,B displays the plot of (F(R)hυ)^2^ against hυ for the Fe_0.5_Mn_0.5_Co_2_O_4_/Fe_2_O_3_ and Mn_0.5_Zn_0.5_Fe_2_O_4_/Fe_2_O_3_ samples, respectively. Therefore, the transitions that were most abundant in the synthesized nanocomposites were direct allowed transitions. The energy gap (E_g_) is determined by extrapolating each graph until (F(R)hν)^2^ equals 0. The energy gap of the Fe_0.5_Mn_0.5_Co_2_O_4_/Fe_2_O_3_ and Mn_0.5_Zn_0.5_Fe_2_O_4_/Fe_2_O_3_ samples was 3.50 and 4.3 eV and 3.52 and 4.20 eV, respectively.

### 3.2. Photocatalytic Degradation of Rhodamine B and Congo Red Dyes

#### 3.2.1. Effect of pH

**Figure 8:**
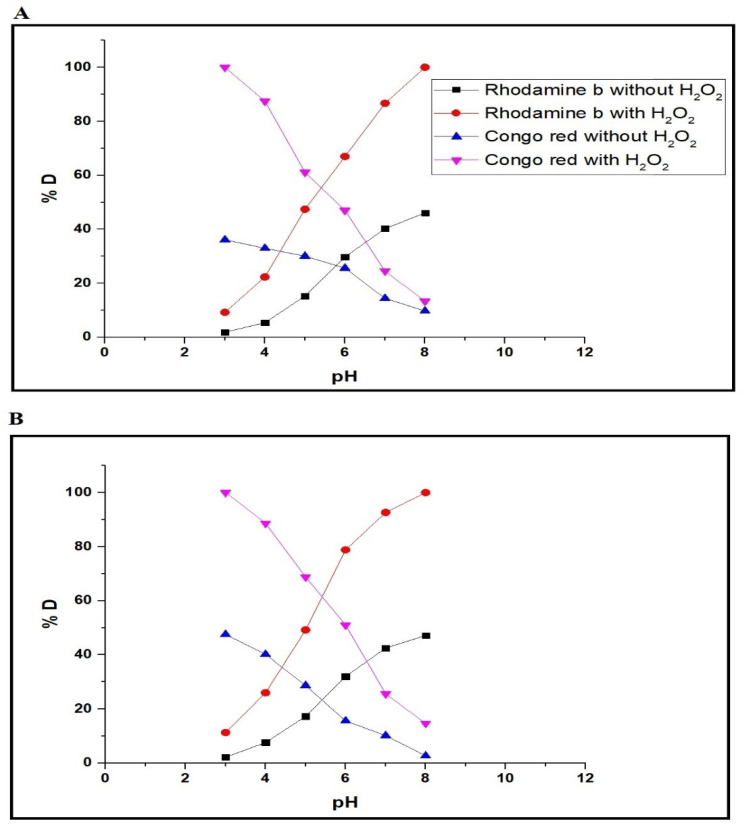
The plot of % D against pH for the degradation of Rhodamine B and Congo Red dyes using the Fe_0.5_Mn_0.5_Co_2_O_4_/Fe_2_O_3_ (**A**) and Mn_0.5_Zn_0.5_Fe_2_O_4_/Fe_2_O_3_ (**B**) samples. Experimental parameters: Concentration of dye = 20 mg/L; Volume of dye = 100 mL; Quantity of catalyst = 0.1 g; UV irradiation time = 100 min.

Figure 8A,B displays the plot of % D against pH for the degradation of Rhodamine B and Congo Red dyes using the Fe_0.5_Mn_0.5_Co_2_O_4_/Fe_2_O_3_ and Mn_0.5_Zn_0.5_Fe_2_O_4_/Fe_2_O_3_ samples, respectively. It is noticeable that by increasing the pH, the degradation efficiency of the synthesized samples toward Rhodamine B dye increased, while the degradation efficiency of the synthesized samples toward Congo Red dye decreased. Rhodamine B dye is a cationic dye whose adsorption is increased in alkaline media, and thus the efficiency of its degradation in alkaline media is increased [19]. Congo Red dye is an anionic dye whose adsorption is increased in acidic media, and thus the efficiency of its degradation in acidic media is increased [19]. In the case of using the Fe_0.5_Mn_0.5_Co_2_O_4_/Fe_2_O_3_ sample, the degradation efficiency of the sample toward Rhodamine B dye without H_2_O_2_ (pH = 8), Rhodamine B dye with H_2_O_2_ (pH = 8), Congo Red dye without H_2_O_2_ (pH = 3), and Congo Red dye with H_2_O_2_ (pH = 3) was equal to 46.02, 100, 36.11, and 100 %, respectively. In the case of using the Mn_0.5_Zn_0.5_Fe_2_O_4_/Fe_2_O_3_ sample, the degradation efficiency of the sample toward Rhodamine B dye without H_2_O_2_ (pH = 8), Rhodamine B dye with H_2_O_2_ (pH = 8), Congo Red dye without H_2_O_2_ (pH = 3), and Congo Red dye with H_2_O_2_ (pH = 3) was equal to 47.14, 100, 47.53, and 100 %, respectively.

#### 3.2.2. Effect of Time

**Figure 9:**
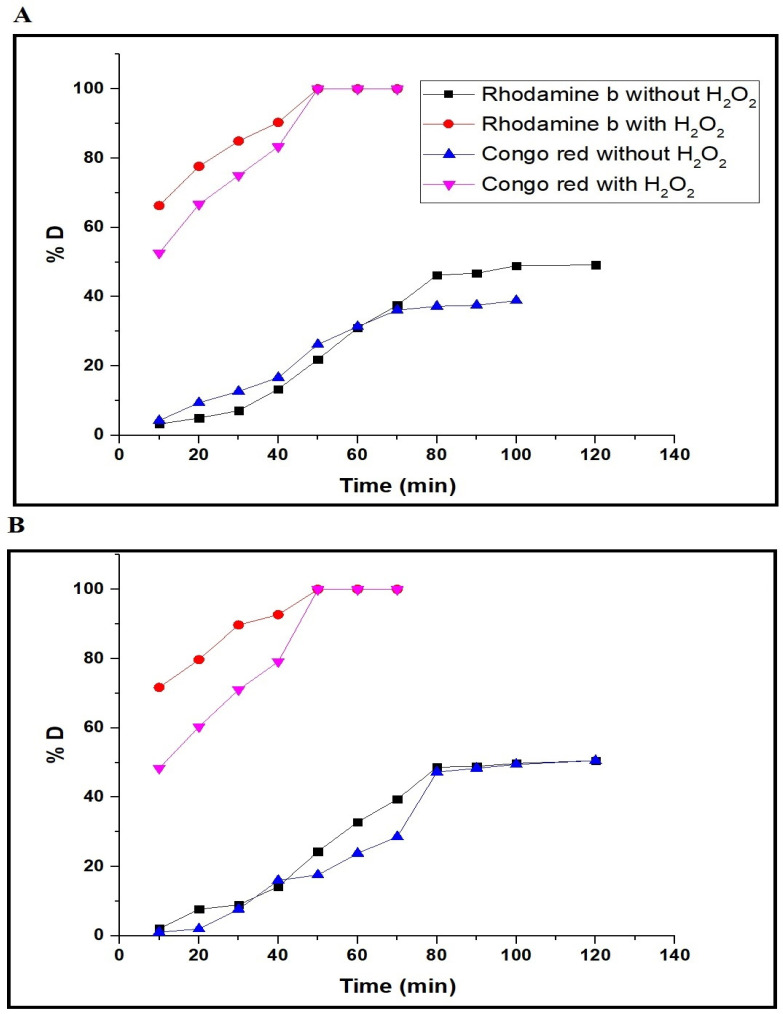
The plot of % D against time for the degradation of Rhodamine B and Congo Red dyes using the Fe_0.5_Mn_0.5_Co_2_O_4_/Fe_2_O_3_ (**A**) and Mn_0.5_Zn_0.5_Fe_2_O_4_/Fe_2_O_3_ (**B**) samples. Experimental parameters: Concentration of dye = 20 mg/L; Volume of dye = 100 mL; Quantity of catalyst = 0.1 g; pH = 8 and 3 in the case of Rhodamine B and Congo Red dyes, respectively.

Figure 9A,B displays the plot of % D against time for the degradation of Rhodamine B and Congo Red dyes using the Fe_0.5_Mn_0.5_Co_2_O_4_/Fe_2_O_3_ and Mn_0.5_Zn_0.5_Fe_2_O_4_/Fe_2_O_3_ samples, respectively. In the absence of hydrogen peroxide, it was observed that by increasing the time from 10 to 80 min, the degradation efficiency of the synthesized samples toward Rhodamine B and Congo Red dyes increased. Additionally, in the case of increasing the time from 80 to 120 min, there was no significant change in the degradation efficiency of the synthesized samples toward Rhodamine B and Congo Red dyes due to the saturation of the active sites of the samples [19]. In the presence of hydrogen peroxide, the complete degradation of Rhodamine B and Congo Red dyes occurred within 50 min. The Rhodamine B and Congo Red dyes were completely degraded under UV light using only hydrogen peroxide in the absence of the synthesized samples within 5 h, which is much larger than the consumed time (50 min) in the presence of the synthesized samples.

**Figure 10:**
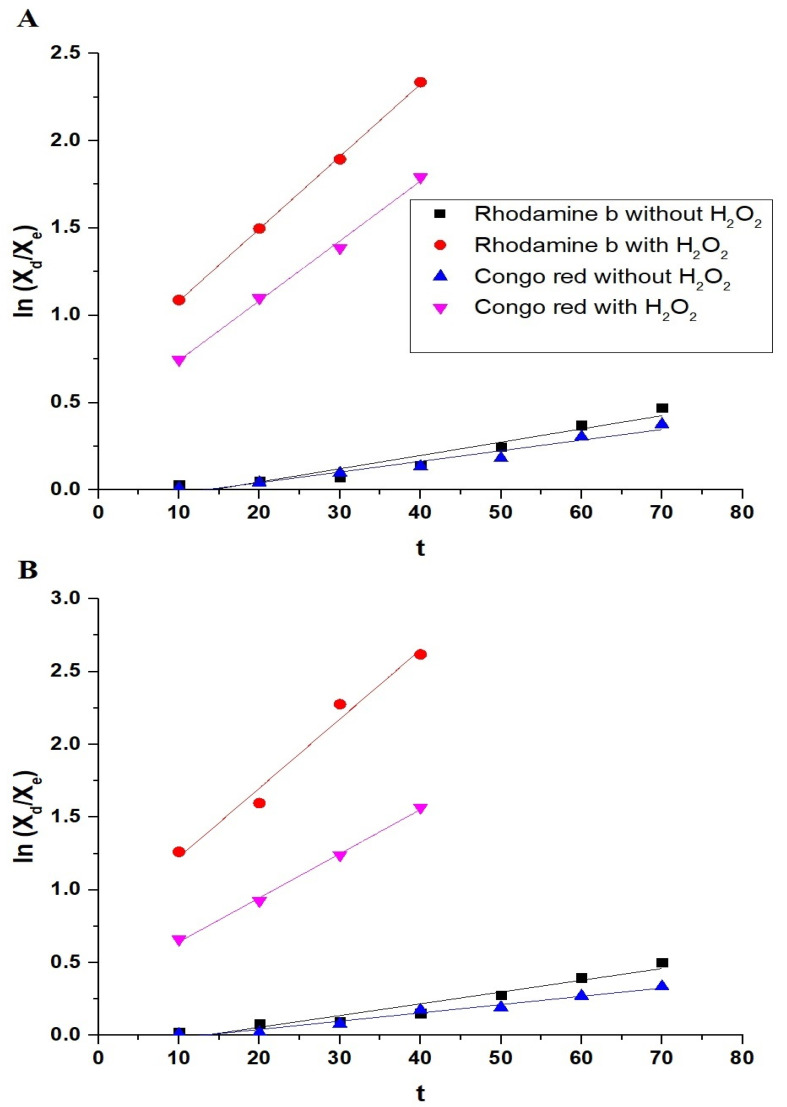
The plot of ln (X_d_/X_e_) against t for the degradation of Rhodamine B and Congo Red dyes using the Fe_0.5_Mn_0.5_Co_2_O_4_/Fe_2_O_3_ (**A**) and Mn_0.5_Zn_0.5_Fe_2_O_4_/Fe_2_O_3_ (**B**) samples.

The degradation of Rhodamine B and Congo Red dyes using the synthesized samples is compatible with the first-order kinetic model as indicated by Equation (3) [19].

ln (X_d_/X_e_) = k t (3)

k (1/min) represents the first-order constant. Figure 10A,B displays the plot of ln (X_d_/X_e_) against t for the degradation of the Rhodamine B and Congo Red dyes using the Fe_0.5_Mn_0.5_Co_2_O_4_/Fe_2_O_3_ and Mn_0.5_Zn_0.5_Fe_2_O_4_/Fe_2_O_3_ samples, respectively. Table 3 and Table 4 display the values for k and R^2^ in the case of using the Fe_0.5_Mn_0.5_Co_2_O_4_/Fe_2_O_3_ and Mn_0.5_Zn_0.5_Fe_2_O_4_/Fe_2_O_3_ samples, respectively. Hydrogen peroxide increases the efficiency of dye degradation, and thus the k value increases when using hydrogen peroxide compared to when it is absent.

#### 3.2.3. Effect of Quantity of Catalyst

**Figure 11:**
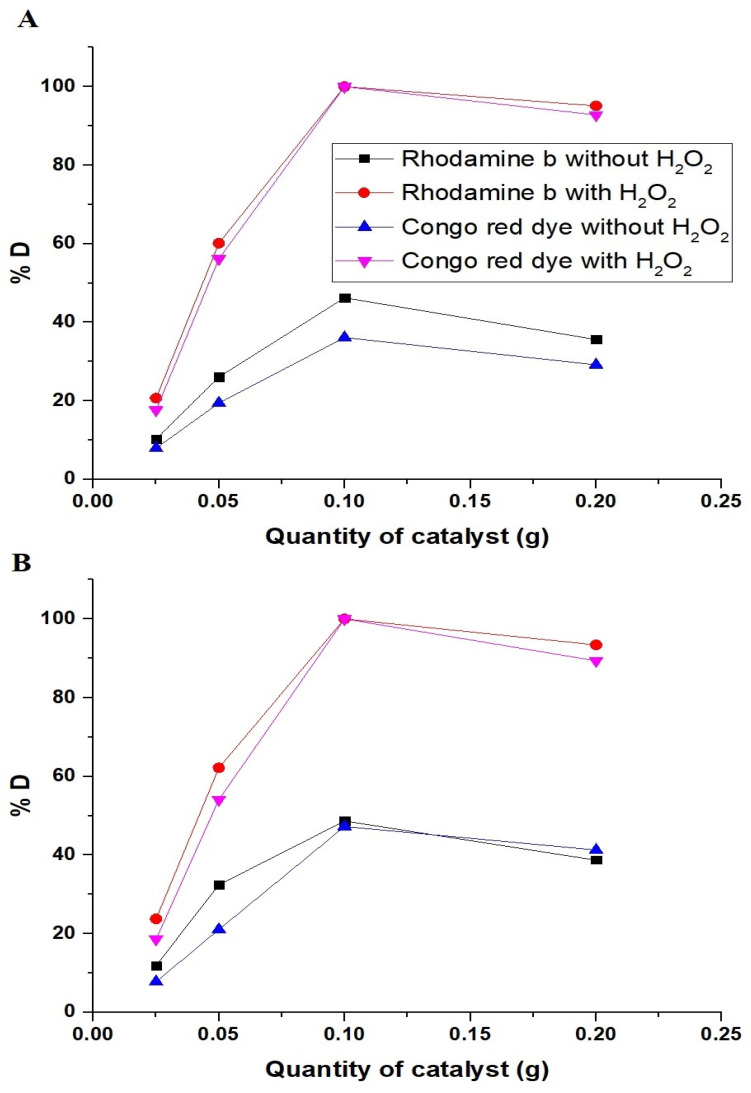
The plot of % D against the quantity of the Fe_0.5_Mn_0.5_Co_2_O_4_/Fe_2_O_3_ (**A**) and Mn_0.5_Zn_0.5_Fe_2_O_4_/Fe_2_O_3_ (**B**) samples for the degradation of Rhodamine B and Congo Red dyes. Experimental parameters: Concentration of dye = 20 mg/L; Volume of dye = 100 mL; pH = 8 and 3 in the case of Rhodamine B and Congo Red dyes, respectively. Time = 80 and 50 min in the absence and presence of H_2_O_2_, respectively.

Figure 11A,B displays the plot of % D against the quantity of the Fe_0.5_Mn_0.5_Co_2_O_4_/Fe_2_O_3_ and Mn_0.5_Zn_0.5_Fe_2_O_4_/Fe_2_O_3_ samples for the degradation of the Rhodamine B and Congo Red dyes, respectively. It has been observed that by increasing the quantity of samples from 0.025 to 0.1 g, the degradation efficiency of the synthesized samples toward Rhodamine B and Congo Red dyes increases because of the increase in active sites [19]. Additionally, when the quantity of the samples was increased from 0.1 to 0.2 g, there was a significant decrease in the degradation efficiency of the synthesized samples toward Rhodamine B and Congo Red dyes because of the turbidity caused by the particles of the catalyst, which impedes the arrival of light to it [19].

#### 3.2.4. Effect of Concentration

**Figure 12:**
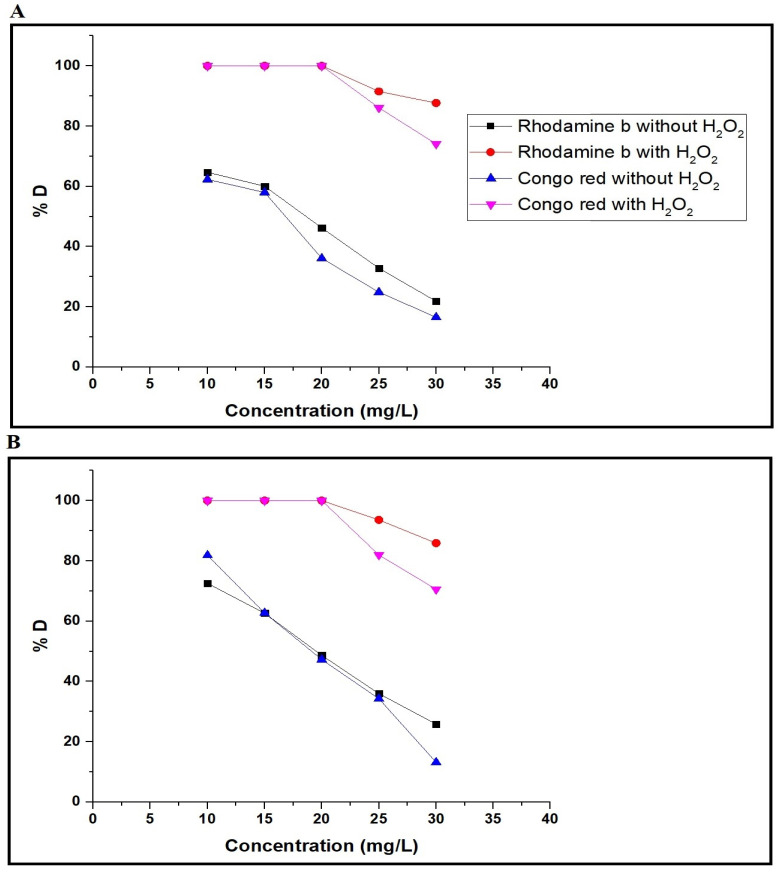
The plot of % D against the concentration of Rhodamine B and Congo Red dyes using the Fe_0.5_Mn_0.5_Co_2_O_4_/Fe_2_O_3_ (**A**) and Mn_0.5_Zn_0.5_Fe_2_O_4_/Fe_2_O_3_ (**B**) samples. Experimental parameters: Volume of dye = 100 mL; Quantity of catalyst = 0.1 g; pH = 8 and 3 in the case of Rhodamine B and Congo Red dyes, respectively. Time = 80 and 50 min in the absence and presence of H_2_O_2_, respectively.

Figure 12A,B displays the plot of the % D of the Rhodamine B and Congo Red dyes against the concentration of the dyes, using the Fe_0.5_Mn_0.5_Co_2_O_4_/Fe_2_O_3_ and Mn_0.5_Zn_0.5_Fe_2_O_4_/Fe_2_O_3_ samples, respectively. It has been observed that by increasing the concentration of the Rhodamine B and Congo Red dyes from 10 to 30 mg/L, the degradation efficiency of the synthesized nanocomposites toward the Rhodamine B and Congo Red dyes decreases because the high concentration makes the dye particles block light from reaching the samples [19].

#### 3.2.5. Effect of Reusability

**Figure 13:**
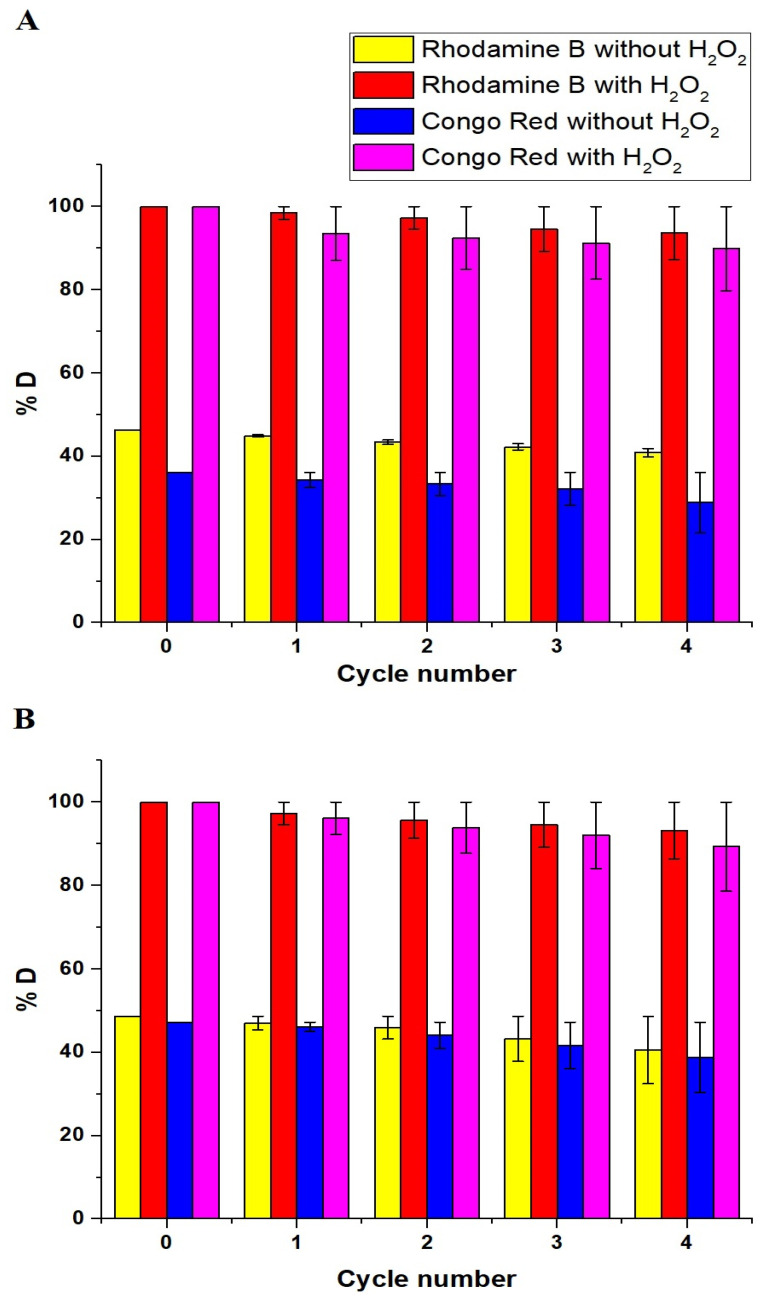
The plot of % D against cycle number for the degradation of Rhodamine B and Congo Red dyes using the Fe_0.5_Mn_0.5_Co_2_O_4_/Fe_2_O_3_ (**A**) and Mn_0.5_Zn_0.5_Fe_2_O_4_/Fe_2_O_3_ (**B**) samples.

Figure 13A,B displays the plot of % D against the cycle number for the degradation of the Rhodamine B and Congo Red dyes using the Fe_0.5_Mn_0.5_Co_2_O_4_/Fe_2_O_3_ and Mn_0.5_Zn_0.5_Fe_2_O_4_/Fe_2_O_3_ samples, respectively. The results demonstrate a minor variation in the value of % D after four cycles, confirming the efficacy of the synthesized samples and their reusability with nearly the same efficiency in degrading the Rhodamine B and Congo Red dyes.

### 3.3. Mechanism of Photocatalytic Degradation

**Figure 14:**
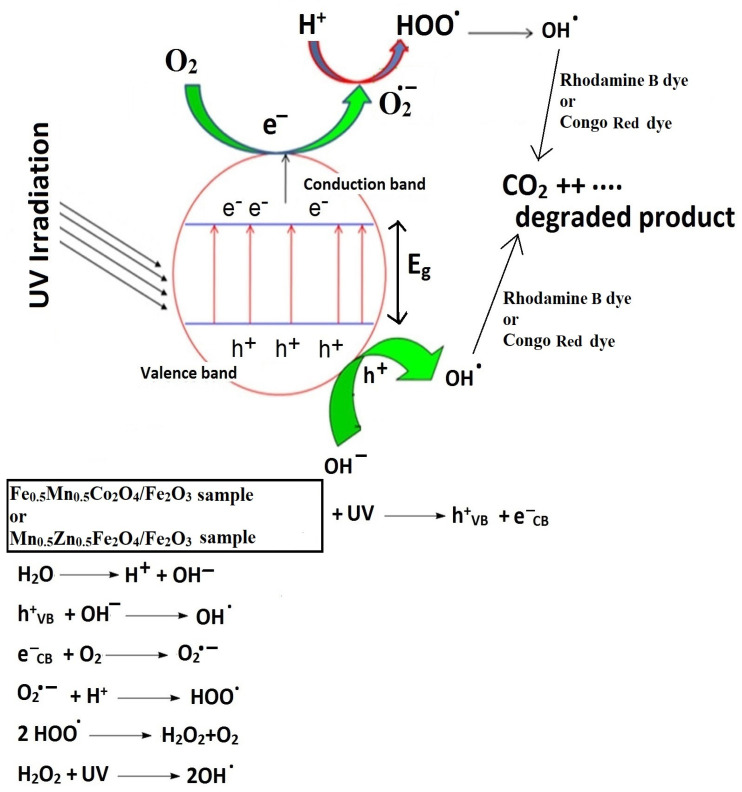
The suggested mechanism for the degradation of Rhodamine B and Congo Red dyes using the Fe_0.5_Mn_0.5_Co_2_O_4_/Fe_2_O_3_ and Mn_0.5_Zn_0.5_Fe_2_O_4_/Fe_2_O_3_ samples.

Figure 14 displays the suggested mechanism for the degradation of Rhodamine B and Congo Red dyes using the Fe_0.5_Mn_0.5_Co_2_O_4_/Fe_2_O_3_ and Mn_0.5_Zn_0.5_Fe_2_O_4_/Fe_2_O_3_ samples. The absorption of photons by a photocatalyst results in the transmission of some electrons from the valence band to the conduction band. Hence, this simultaneously generates electrons and holes in the conduction and valence bands, respectively. Electrons and holes can produce hydroxyl free radicals when reacting with water. Rhodamine B and Congo Red dyes can be quickly degraded by hydroxyl free radicals and converted into volatile gases, such as CO_2_ and H_2_O [19,20].

### 3.4. A Comparison between the Synthesized Nanocomposites and other Catalysts in the Literature for the Degradation of Rhodamine B and Congo Red Dyes

The % D of the Rhodamine B dye utilizing the Fe_0.5_Mn_0.5_Co_2_O_4_/Fe_2_O_3_ and Mn_0.5_Zn_0.5_Fe_2_O_4_/Fe_2_O_3_ samples was compared to that of other catalysts used in earlier studies, including the ZnO/SnO_2_ composite, Fe/SnO_2_ composite, Fe_3_O_4_/TiO_2_/CoMoO_4_ composite, Fe_3_O_4_/TiO_2_ composite, chitosan/SnO_2_ composite, Fe_3_O_4_/SiO_2_/TiO_2_ composite, BiOI/BiOCl composite, and ZnO/PbCrO_4_ composite as displayed in Table 5 [41,42,43,44,45,46,47,48]. The % D of the Congo Red dye utilizing the Fe_0.5_Mn_0.5_Co_2_O_4_/Fe_2_O_3_ and Mn_0.5_Zn_0.5_Fe_2_O_4_/Fe_2_O_3_ samples was compared to that of other catalysts used in earlier studies, including the ZrO_2_/CeO_2_/ZnO, Au/ZnO, Ag/ZnO, magnetic silica-coated Ag_2_WO_4_/Ag_2_S, and TiO_2_-doped cobalt ferrite as displayed in Table 6 [49,50,51,52]. The results demonstrate the photocatalytic superiority of the Fe_0.5_Mn_0.5_Co_2_O_4_/Fe_2_O_3_ and Mn_0.5_Zn_0.5_Fe_2_O_4_/Fe_2_O_3_ samples over other photocatalysts used in previous studies because they degrade a large volume of a high concentration of Rhodamine B and Congo Red dyes in a short period of time with high efficiency. Thus, these synthesized catalysts are joined to a series of active materials for the degradation of organic materials [53,54]. Feng et al. utilized a new dual-mode-driven micromotor based on foam-like carbon nitride (f-C_3_N_4_) with precipitated Fe_3_O_4_ nanoparticles, namely Fe_3_O_4_/f-C_3_N_4_, powered by chemical/magnetic stimuli for a rapid reduction in organic pollutants [55]. Li et al. prepared an ordered Schottky heterojunction of heptazine-based crystalline carbon nitride (HCN) and Ti_3_C_2_ MXene through the ionothermal method. The HCN/Ti_3_C_2_ composites exhibit higher photocatalytic performance than pristine HCN [56].

## 4. Conclusions

The Pechini sol–gel technique was employed for the facile synthesis of Mn_0.5_Zn_0.5_Fe_2_O_4_/Fe_2_O_3_ and Fe_0.5_Mn_0.5_Co_2_O_4_/Fe_2_O_3_ as mixed metal oxide nanoparticles for the efficient photocatalytic degradation of Rhodamine B and Congo Red dyes. The XRD patterns revealed that the average crystallite size of the Fe_0.5_Mn_0.5_Co_2_O_4_/Fe_2_O_3_ and Mn_0.5_Zn_0.5_Fe_2_O_4_/Fe_2_O_3_ samples was 90.25 and 80.62 nm, respectively. In the presence of hydrogen peroxide, the complete degradation of 100 mL of 20 mg/L of Rhodamine B and Congo Red dyes occurred at pH = 8 and 3, respectively, within 50 min and using 0.1 g of the synthesized samples.

## Figures and Tables

**Table 1:** The weight percentages of the elements in the synthesized samples.

Elements	Wt %	
	Fe_0.5_Mn_0.5_Co_2_O_4_/Fe_2_O_3_	Mn_0.5_Zn_0.5_Fe_2_O_4_/Fe_2_O_3_
Fe	75.10	69.51
Mn	6.06	4.52
Co	8.24	----
Zn	----	10.29
O	10.60	15.68

**Table 2:** The BET surface area, total pore volume, and average pore size of the synthesized samples.

Surface Properties	Sample	
	Mn_0.5_Zn_0.5_Fe_2_O_4_/Fe_2_O_3_	Fe_0.5_Mn_0.5_Co_2_O_4_/Fe_2_O_3_
BET surface area (m^2^/g)	62.7305	44.2141
Total pore volume (cc/g)	0.0490	0.0925
Average pore size (nm)	1.7235	3.2579

**Table 3:** The k and R^2^ values for the degradation of Rhodamine B and Congo Red dyes using the Fe_0.5_Mn_0.5_Co_2_O_4_/Fe_2_O_3_ sample.

Dye	k (1/min)		R^2^	
	With H_2_O_2_	Without H_2_O_2_	With H_2_O_2_	Without H_2_O_2_
Rhodamine B dye	0.0076	0.0414	0.9139	0.9993
Congo Red dye	0.0061	0.0342	0.9479	0.9939

**Table 4:** The k and R^2^ values for the degradation of Rhodamine B and Congo Red dyes using the Mn_0.5_Zn_0.5_Fe_2_O_4_/Fe_2_O_3_ sample.

Dye	k (1/min)		R^2^	
	With H_2_O_2_	Without H_2_O_2_	With H_2_O_2_	Without H_2_O_2_
Rhodamine B dye	0.0081	0.0475	0.0057	0.0303
Congo Red dye	0.9318	0.9695	0.9696	0.9965

**Table 5:** Comparison between the photocatalytic activities of the Fe_0.5_Mn_0.5_Co_2_O_4_/Fe_2_O_3_ and Mn_0.5_Zn_0.5_Fe_2_O_4_/Fe_2_O_3_ samples and those of other catalysts in earlier studies toward the Rhodamine B dye.

Catalyst	Concentration of Dye (mg/L)	Volume of Dye (mL)	Amount of Catalyst (g)	% D	Time (min)	Ref
BiOI/BiOCl composite	5	50	0.025	99.2	60	[41]
ZnO/PbCrO_4_ composite	4.79	100	0.1	95	60	[42]
Chitosan/SnO_2_ composite	4.79	100	0.05	95	60	[43]
Fe_3_O_4_/SiO_2_/TiO_2_ composite	30	50	0.05	29.5	60	[44]
Fe_3_O_4_/TiO_2_/CoMoO_4_ composite	20	50	0.05	98.7	17	[45]
Fe_3_O_4_/TiO_2_ composite	10	100	0.05	91	120	[46]
ZnO/SnO_2_ composite	0.958	50	0.05	80	120	[47]
Fe/SnO_2_ composite	10	50	0.025	55	120	[48]
Fe_0.5_Mn_0.5_Co_2_O_4_/Fe_2_O_3_ composite	20	100	0.1	100	50	This study
Mn_0.5_Zn_0.5_Fe_2_O_4_/Fe_2_O_3_ nanocomposite	20	100	0.1	100	50	This study

**Table 6:** Comparison between the photocatalytic activities of the Fe_0.5_Mn_0.5_Co_2_O_4_/Fe_2_O_3_ and Mn_0.5_Zn_0.5_Fe_2_O_4_/Fe_2_O_3_ samples and those of other catalysts in earlier studies toward the Congo Red dye.

Catalyst	Concentration of Dye (mg/L)	Volume of Dye (mL)	Amount of Catalyst (g)	% D	Time (min)	Ref
ZrO_2_/CeO_2_/ZnO	10	100	0.005	86	250	[49]
Au/ZnO	16	100	0.05	77.2	150	[50]
Ag/ZnO	16	100	0.05	81.6	150	[50]
Magnetic silica-coated Ag_2_WO_4_/Ag_2_S	20	100	0.1	99.5	140	[51]
TiO_2_-doped cobalt ferrite	10	100	0.08	85	120	[52]
Fe_0.5_Mn_0.5_Co_2_O_4_/Fe_2_O_3_ composite	20	100	0.1	100	50	This study
Mn_0.5_Zn_0.5_Fe_2_O_4_/Fe_2_O_3_ nanocomposite	20	100	0.1	100	50	This study

## Data Availability

Not applicable.
